# Discrete Global Grid Systems as scalable geospatial frameworks for characterizing coastal environments

**DOI:** 10.1016/j.envsoft.2021.105210

**Published:** 2021-12-01

**Authors:** Justin Bousquin

**Affiliations:** Gulf Ecosystem Measurement and Modeling Division, U.S. Environmental Protection Agency, Office of Research and Development, Center for Environmental Measurement and Modeling, Gulf Breeze, FL, USA

**Keywords:** Discrete Global Grid System, Coastal, Water quality, Geospatial framework, Big data

## Abstract

Data portals and services have increased coastal water quality data availability and accessibility. However, tools to process this data are limited – geospatial frameworks at the land-sea interface are either adapted from open- water frameworks or extended from watershed frameworks. This study explores use of a geospatial framework based on hexagons from a Discrete Global Grid System (DGGS) in a coastal area. Two DGGS implementations are explored, dggridR and H3. The geospatial frameworks are compared based on their ability to aggregate data to scales from existing frameworks, integrate data across frameworks, and connect flows across the land-sea interface. dggridR was simpler with more flexibility to match scales and use smaller units. H3 was more performant, identifying neighbors and moving between scales more efficiently. Point, line and grid data were aggregated to H3 units to test the implementation’s ability to model and visualize coastal data. H3 performed these additional tasks well.

## 1. Introduction

People live, work, and recreate in coastal waters, making the quality of those waters pivotal to our health, society, and economy. A number of organizations, including state and federal regulatory agencies, monitor a variety of coastal water quality characteristics (e.g., temperature, dissolved oxygen, total suspended solids, fecal coliform, chlorophyll content, nutrients, etc.). Water quality data are used to characterize waters, identify trends over time, identify emerging problems, determine effectiveness of restoration or pollution controls, help direct restoration and pollution control efforts to where they are most needed, and respond to emergencies such as floods or spills (USEPA, 2013). These uses often require water quality data at different scales; for example, siting a restoration project requires data that can differentiate two neighboring sites along the same shoreline, whereas a state impairments assessment requires data that can differentiate bays within an estuary, whereas the EPA’s National Coastal Condition Assessment (NCCA) differentiates condition in one estuary compared to another (USEPA, 2001). Such use cases all have differences in spatial and temporal scales, quality assurance thresholds, geographic locality, etc.; but one commonality is that most users need to depict or relate water quality spatially using geospatial frameworks. A geospatial framework using a new grid system to aggregate, integrate and scale water quality data will add benefit, particularly for ecological applications in coastal areas made more complicated by the land/water interface.

The volume of available water quality data is skyrocketing with the development of ocean-observing arrays and in-situ buoys (Glasgow et al., 2004; Legler et al., 2015), interconnected low cost sensors (Adamo et al., 2015; Park et al., 2020; Vijayakumar and Ramya, 2015), and inference methods for remote sensing (Gholizadeh et al., 2016; IOGC, 2018; Ross et al., 2019). Portals and web services help to standardize and share this water quality data (Blodgett et al., 2016). For example, the Water Quality Exchange (WQX) allows sharing of data using a standardized data model and the Water Quality Portal (WQP) provides access to that data (Read et al., 2017). However, each such data collection is best suited for data derived a certain way. For example, the Consortium of Universities for the Advancement of Hydrologic Sciences (CUAHSI; Geosling et al., 2015) Hydrologic Information System may be more appropriate than WQP for real-time continuous data streams. To analyze this wealth of water quality data, the data must be gathered from various portals and services, harmonized and integrated into one spatial framework.

Most end users need to depict or relate water quality spatially, and often across the land/water interface. Geospatial frameworks help to structure these data for use in analysis. Historically, geospatial data for onshore and offshore areas are not well integrated due to fundamental differences in mapping conventions and reference datums (National Research Council, 2004). NOAA has developed methods to consistently define the tidal datum for most of the nation, allowing water level-based shoreline definitions to be related to other vertical datums (Parker et al., 2003). However, once the shoreline is defined by water level, keeping it up to date is complicated by the dynamic nature of shorelines which move over time due to erosion and accretion (Boak and Turner, 2005; Vos et al., 2019).

Several geospatial frameworks exist for coastal areas, with both consistencies and differences in how units are defined, how they deal with shorelines and how far to extend offshore. The National Hydrography Dataset Plus (NHDPlus) medium resolution uses catchments to define the area of the landscape that drains to a specific stream segment (Moore and Dewald, 2016). Linkages and consistencies between NHDPlus catchments and other frameworks or datasets have made it easier to standardize and integrate information. State regulatory waterbody units used state-specific boundaries that may or may not include coastline definitions, but EPA’s Assessment, Total Maximum Daily Load (TMDL) Tracking and Implementation System (ATTAINS) geospatial framework standardizes assessment units using NHDPlus (USEPA, 2016a). The WQP indexes sample sites to the NHDPlus catchments, utilizing the upstream-downstream relational network to define hydrologic relationships and hierarchies (Read et al., 2017). When NHDPlus catchments were delineated they were related to a snapshot of the Watershed Boundary Dataset (WBD; Berelson et al., 2004) at Hydrologic Unit Code (HUC) 8 and 12 (McKay et al., 2012). However, relating the datasets does not mean the boundary definitions were completely aligned, and WBD units have been updated since making it difficult to relate current units. WBD units typically extend out farther into coastal areas than land catchments and larger HUC units use the NOAA three nautical mile line as a default offshore boundary (USGS, 2013). The NHDPlus framework uses National Elevation Dataset (NED) elevations to define catchment boundaries, meaning these align with a consistent 30 × 30-m square grid. However, catchment boundaries only extended out into coastal areas as far as elevation models can direct flows (i.e. stopping where elevation is zero), resulting in catchments that excluded shorelines and estuary boundaries, and inhibited networking coastal sample sites to upstream conditions. NHDPlus recently added ocean catchments, a 1500 × 1500-m grid that extends seamlessly from land catchments out to the NOAA 14-mile Maritime Limit (USGS, 2019). The NED system has now been superseded by the 3D Elevation Program (3DEP), part of the coastal national elevation database (CoNED; Danielson et al., 2018) and national map (Arundel et al., 2015). The NHDPlus High Resolution dataset in development uses 3DEP elevations and differentiates estuaries from the ocean (Moore et al., 2019), suggesting coastline definitions in the medium resolution land and ocean catchments may evolve.

Although square grids are widely adopted as the basis for geospatial frameworks, one viable alternative is hexagonal grids. Hexagonal grids have several advantages over square grids for ecological applications (Birch et al., 2007). A hexagon unit has a smaller perimeter to diameter ratio than a square unit, meaning it more closely resembles a circle and has less bias from edge effects. This is an important attribute when a point data sample is used to characterize the larger unit as a whole. Hexagons are better for connectivity and path analysis, because the centroids (center points) of all six neighboring hexagons are equidistant (Birch et al., 2007). In contrast, square units have eight neighbors, the four neighbors sharing a side are at one distance whereas the other four, diagonal neighbors sharing a vertex, are at a greater distance.

Hexagonal grids have been used as the basis for coastal frameworks, both for smaller scale habitat assessments (Smith et al., 2010) and for broader scale characterizations (Shamaskin et al., 2020). The 2019 NOAA/Alaska Department of Natural Resources coastal priorities assessment used a hexagon-based framework to gather stakeholder mapping needs for CoNED (Alaska DNR, 2020). EPA’s NCCA (USEPA, 2016b) samples conditions at points selected through Generalized Random Tessellation Stratified survey design. These sampling plans are an extension of the former Environmental Monitoring and Assessment Program (EMAP; Diaz-Ramos et al., 1996), where spatially weighted sample points were chosen from a tessellation of triangles to place hexagon unit centroids. For statistical randomness the sample site selection methods start the hexagonal grid from a random point (Pebesma et al., 2021), but for consistency some applications have instead used the origin point (i.e. min x and min y) of the sample frame or area of interest (e.g. Myers et al., 2021). Tools have generated hexagons to fill the area based on the desired number of sample units, desired hexagon area (e.g. Evans et al., 2021), or a consistent distance between hexagon centroids. These differences in hexagon generation and alignment make it difficult to integrate results from disparate efforts or to promote one as the basis for a coastal spatial framework.

A reasonable alternative to using an arbitrary starting point for the hexagonal grids is to use a Discrete Global Grid System (DGGS). A DGGS is a hierarchical data structure for covering the surface of the earth with a consistent grid of regular shaped, equal area units. In this case, the earth is treated as an isosahedron divided into hexagons. Units spatially reference locations, bypassing transformation issues related to planar representations of coordinates. DGGSs are not a new concept; in fact, they were considered in the early EMAP surveys (Carr et al., 1997). A DGGS provides advantages for Big Data and Digital Earth research; the past decade has seen several DGGS advancements, new implementations, and example applications (Birk, 2018; Sahr, 2011; Sirdeshmukh et al., 2019; Jendryke and McClure, 2019). Several implementations of DGGS have used hexagons as their equal area units, including H3 (Brodsky, 2018), DGGRID (Sahr, 2019a), OpenEager (Bush, 2017), and rHEALPix (Gibb, 2016). DGGSs have become pervasive enough that the Open Geospatial Consortium (OGC) has started developing standards for them (Purss et al., 2017). Although current implementations may not yet adhere to all these standards (Bondaruk et al., 2020), there is a clear advantage to begin integrating these data structures.

Coastal areas can benefit from a new DGGS geospatial framework, but it must function at the necessary scales and integrate with existing geospatial frameworks given their spatial overlap and upstream relationship to coastal areas. This manuscript focuses on two open-source implementations of hexagonal frameworks: H3 (Uber, 2020) and dggridR (Barnes and Sahr, 2017). The methodology behind how the two hexagonal implementations each scale data is described. Hexagon scales are then compared to average units from NHDPlus, the WBD HUC-08, and the regulatory units used in ATTAINS. Next, we show how H3 hexagonal framework units network together and how to integrate that network with NHDPlus land catchments. For comparison, NHDPlus ocean catchments are also networked together and as downstream of land catchments. Lastly, the H3 implementation is used to convert, aggregate, scale and interpolate actual coastal temperature data for data-rich Tampa Bay, Florida. Converted data types included point, line, and remotely sensed grid data.

## 2. Methods

### 2.1 Hexagonal frameworks and scaling

Two open-source implementations of hexagon-based DGGSs were used: the H3 python library v3.6.4 (Uber, 2020) and the dggridR R package (Barnes and Sahr, 2017). Both implementations are available in multiple programing languages. This manuscript’s supplemental materials include raw data and code examples. The code is presented within jupyter notebooks to allow users to repeat our methods on the provided raw data (Kluyver et al., 2016). Although maps in this manuscript highlight example areas, the jupyter notebook allows users to explore results by panning or zooming to other places on the map.

Hexagon projection and orientation are essential to consistent units. H3 hexagons utilize an icosahedral projection with spherical orientation (Fuller, 1975), with a pentagon at each of twelve icosahedron vertices. dggridR is an R package for implementing the DGGRID software (v6.2b; Sahr, 2019a), and supports the same typologies (i.e. hexagons, triangles, or diamonds) and projections (e.g. Icosahedral Snyder Equal Area). To be consistent with H3, all dggridR examples used hexagons, the icosahedral projection, and spherical Fuller orientation.

**Fig. 1.**
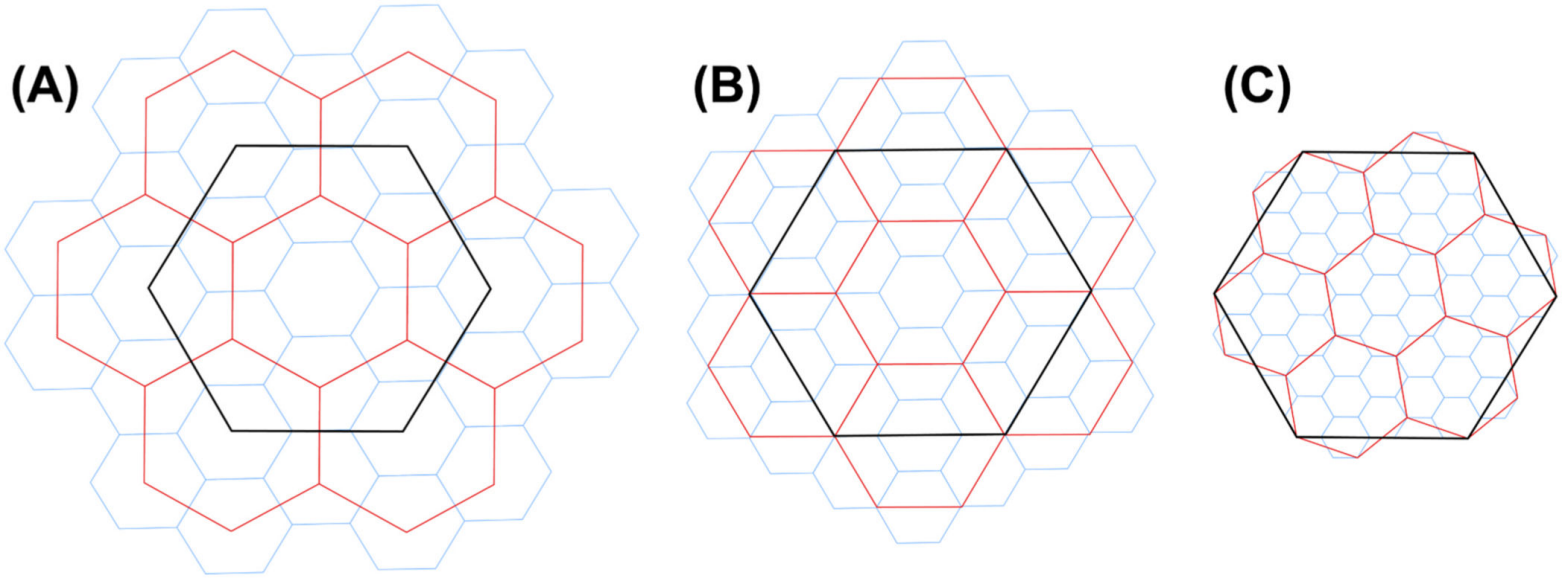
Hierarchical hexagon grid resolutions scaling using (A) aperture-3, (B) aperture-4, and (C) aperture-7. dggridR generated the grids for apertures 3 and 4, H3 generated grids for aperture-7.

Hexagonal DGGS scaling involves moving from a finer resolution “child” hexagon, to a coarser resolution “parent” hexagon, or vice-versa. One of the disadvantages of hexagons as compared to square units is that hexagons are non-congruent between scales. A square unit divides into four, equal-area subunits (e.g. four 30 m × 30 m squares completely fit into one 60 m × 60 m square. H3 and dggridR divide hexagons into seven sub-unit hexagons; but those units either do not completely cover the original hexagon or they exceed the original hexagon bounds, depending on the “aperture” used (i.e., the ratio of child units to a parent unit area). Common apertures used in hexagonal grids are 3, 4 and 7; the smaller the aperture, the smaller the change in unit size from one scale to the next (Fig. 1). In all three apertures, there are seven child units for each parent unit, with the central unit sharing the centroid of the parent unit, but with different ratios of parent to child area and with different orientations (Table 1).

For aperture-3, the centroid of each of the six child units align with the six parent vertices. Rotation of units 90° from one scale to the next results in a change in unit orientation (pointy topped vs. flat topped). A single child unit may overlap as many as three different parent units using aperture 3. Of the three apertures, this aperture has the smallest change in area from one resolution to the next. Aperture-3 is available in dggridR but was not demonstrated here as it is like dggridR aperture-4 in functionality.

For aperture-4, the centerlines of each of six child units align with the six parent edges. The units have the same orientation across the different scales. Aperture-4 has the advantage of splitting each of the six child units evenly between parent units. dggridR can use aperture-3, aperture-4, or mix the two, either to alternate or to approximate a desired unit area at a specific resolution. All dggridR applications in this manuscript used aperture-4 scaling.

Aperture-7 has the advantage of fitting the child units most closely to the area covered by the parent unit. Using this aperture allows H3 to scale data without re-indexing the original coordinates. However, unlike aperture-3 and −4 child units, aperture-7 child units do not completely cover the area of the parent unit. To resample point data at a smaller scale would require consideration of 12 child units instead of six. Section 3.4 compares point data scaled using the index method to re-aggregated data to assess scaling distortion. All H3 applications in this manuscript used aperture-7 scaling.

### 2.2 Scale-matching with existing geospatial frameworks

Hexagon scales must be close to decision scales to be useful for representing information in support of those decisions. To match hexagonal unit scales to decision scales, the hexagon scale with the average hexagon unit area closest to the average unit of three existing frameworks was identified (Table 2). The frameworks considered were the NHDPlus catchments for local decisions (e.g. for restoration), the regulatory units used ATTAINS for sub-estuary decisions (e.g. State impairments), and the WBD HUC-08 for estuary decisions (e.g. NCCA comparisons).

Matching hexagon unit areas to the average unit area of these frameworks helps to identify comparable scales for visualizations but does not capture the information the irregularly shaped units of those frameworks represent. For example, NHDPlus land catchments represent hydrologically connected parts of the landscape because their boundaries are defined by the area of the landscape that drains to a specific NHD stream segment. To appropriately represent hydrologic relationships using the hexagonal frameworks, hexagon units would need to be the same size as the units used to originally delineate the catchments and be related to NHD stream segments. A fourth scale of hexagon units, an elevation data scale, was approximated to match the 30 m grid used to delineate catchments.

NHDPlus land catchments have an average area of 2.9 km^2^. NHDPlus ocean catchments are 1500 × 1500-m square units, but at the land/ ocean catchment boundary these squares are only partial to avoid overlapping land catchments; as a result, the average area for ocean catchments is 2.01 km^2^ (USGS, 2019). ATTAINS waterbodies vary, but the average area is 22 km^2^ (USEPA, 2020). The average WBD 8-digit HUC is 1800 km^2^, with a range of 10.6–19,200 km^2^. The elevation data scale is the smallest square cell considered, 900 m^2^. These square units are regularly shaped and, unlike the other scales, their accuracy is as important as their area. Grid cell accuracy is the maximum distance between any two points in that cell, measured as the distance from vertex to opposite vertex, approximately 42 m. These average areas represent decision scales to approximate with hexagonal units from H3 and dggridR.

### 2.3 Networking units within and across frameworks

Coastal waterbodies are not closed systems; water flows in from upstream watersheds, and mixes within the embayment and with ocean waters outside the embayment. Modeling this mixing is beyond the scope of this manuscript. However, a coastal geospatial framework must have the capacity to network units within the framework to support mixing flows and be able to network as downstream of watershed units.

All the frameworks examined in this manuscript use a unique index to identify individual units. In NHDPlus, each land and ocean catchment geometry has a unique ID. For land catchments, this same ID is stored in a field (COMID) in a separate PlusFlow table that captures the network of upstream/downstream relationships. Flows between units in the estuary are not as easy to define as those in watersheds; but methods using unit indexes are demonstrated for networking ocean catchment and H3 hexagon units, to both neighboring units within those frameworks and to neighboring land catchments.

Ocean catchments and H3 hexagons downstream of land catchments were identified and integrated into the flow network. Downstream ocean catchments were those adjacent to a land catchment, while downstream hexagons were those overlapping a land catchment. The ocean catchment COMIDs or hexagon indices from the corresponding downstream unit were added to the PlusFlow table. Ocean catchments already have a 9-digit index (starting with 7) unique from any land catchment (9-digit land catchment indexes start with either 1 or 9). The H3 index is an unsigned 64-bit integer (uint64; e.g. ‘8b441a8f028cfff’) that had to be converted to store as integer-based indexes in the PlusFlow table (e.g. 627198441950859263). Converted H3 indexes all have more digits than the 9-digit NHDPlus indexes, making it easy to avoid duplicates.

Open water ocean catchments and H3 spatial units, non-adjacent and non-overlapping with land catchments, interconnect with one another as well. Each open water ocean catchment had four neighboring catchments added to the PlusFlow table as downstream. This resulted in all ocean catchments being both upstream and downstream of their neighbors. H3 uses a hierarchical index, where each identifier is specific to a specific spatial unit and resolution (Brodsky, 2018). The index system allows the coarser resolution parent hexagon to be identified from the finer resolution child hexagon by simply truncating the index. The assignment of digits 0–6 at each resolution uses a central place indexing arrangement (Sahr, 2019b), allowing H3 to identify neighboring edge cells, or cells *n* given cell widths away from a unit by its index. H3 has built-in index-based methods (e.g. hexRange and kRing methods) that were used to network units within the framework. Since the center of each hexagon is a consistent distance from its neighbor, these same methods were also used for distance-based interpolation as described in section 2.5.

Compared to H3, dggridR indexing is simpler; each spatial unit in a grid has a global_sequence parameter SEQNUM, numbered 1 to *n*, where *n* is the number of cells generated. However, ddgridR does not have the same built-in index-based neighbor identification as H3 and therefore was not used to network cells within the framework. As with H3 hexagons, any dggridR hexagons overlapping a land catchment could be networked as downstream of that catchment by adding its index to the PlusFlow table. As with ocean catchments, dggridR hexagon neighbor-to-neighbor relationships could be stored by index in the PlusFlow table. In both cases the hexagon index must not duplicate any existing catchment FEATUREID.

### 2.4 Demonstration site and data aggregation

**Fig. 2.**
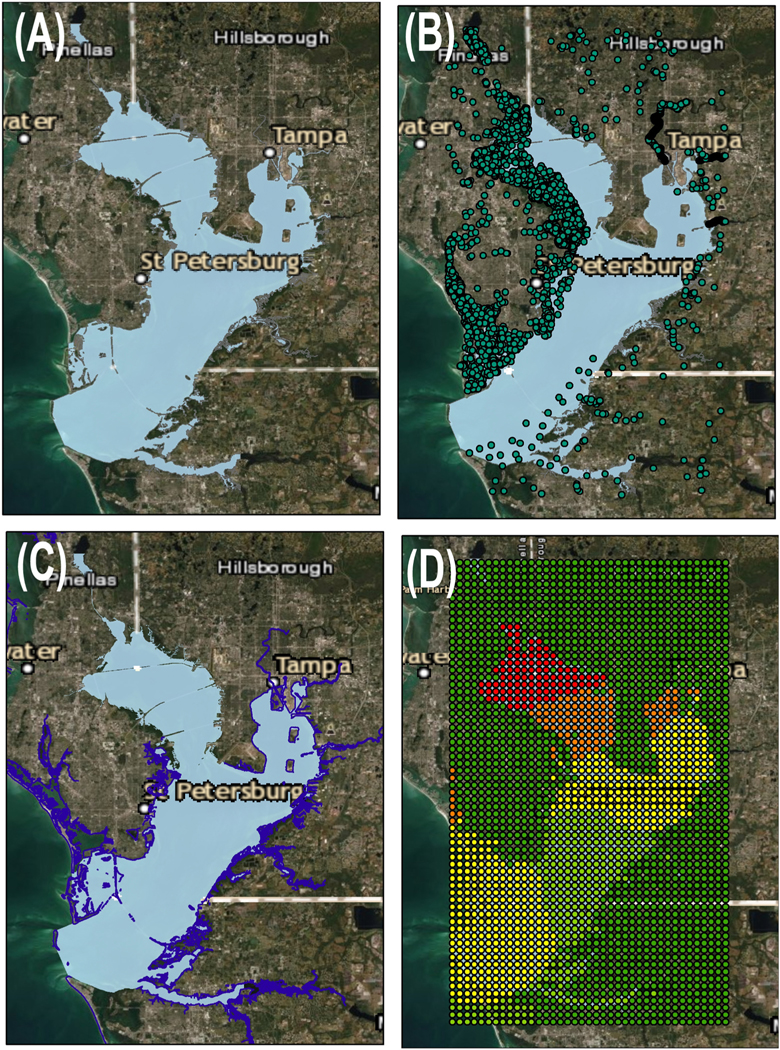
Tampa Bay as defined by NCCA (A). Raw – (B) Point – water quality sites, (C) Line – continually updated shoreline product, and (D) gridded data – remote sensed sea surface temperatures.

Raw data aggregation, data scaling, and integration with NHDPlus using dggridR Hex-aperture-4 and H3 Hex Aperture-7 hexagons are demonstrated in the Tampa Bay estuary as defined by the NCCA survey program (Fig. 2A). Tampa Bay was chosen because the area is data rich. Three datasets – point, line and grid – were converted to H3 hexagon units. The point data came from sampling points in the WQP (Fig. 2B). Results at these sites represented water temperatures uploaded to the WQP (Read et al., 2017; USEPA et al., 2021). Line data came from the NOAA Continually Updated Shoreline Product (CUSP; Fig. 2C). NOAA’s CUSP dataset was downloaded for Gulf of Mexico (NOAA, 2020) and lines intersecting hexagons were characterized as having shoreline present/absent. Grid data came from the Multi-Scale Ultra High Resolution (MUR; Fig. 2D) sea surface temperature dataset, a daily 1 km resolution dataset that merges multiple Level-2 satellite datasets (Chin et al., 2017). Sea Surface temperatures over land were represented with No Data.

Temperature point and grid data were used because it influences species composition, varies more in coastal environments, and correlates with circulation (Azmi et al., 2015; Brando et al., 2015). Physical characteristics, such as Temperature, are also the most common result in WQP (Read et al., 2017).

Point data from the WQP were pre-processed before aggregation to ensure consistent data. The query restricted results to water temperature sample points within the NCCA Tampa Bay polygon extent and from January 1, 1995 through December 31, 2020. Python scripts automated data gathering and pre-processing (see supplemental materials). Pre- processing involved a few key assumptions about coordinates, time zones, depth, and duplicates. Coordinates retrieved for sample sites were transformed to NAD 1983 if in a different datum (e.g. WGS, 1984). If the coordinate reference system was not specified, the script assumed site coordinates were NAD 1983 because WQP uses it in spatial queries. Time zone handling assumed time zone entries were accurate, where any time zone listed as standard time or daylight time uses that Coordinated Universal Time (UTC) adjustment regardless of time of year. For example, Eastern Standard Time (EST) is interpreted as UTC – 5 h and Eastern Daylight Time (EDT) as UTC – 4 h, even if an EST time is during daylight savings. This is consistent with USGS dataRetrieval packages for R or python (De Cicco et al., 2018; Hodson and Black, 2020). Only surface temperatures were examined to be more consistent with sea surface temperatures from remote sensing. Results with a depth greater than 5 m were rejected and those with no depth measurement were assumed to be surface measurements. Only the first of any duplicate temperature for the same location and time was considered.

### 2.5 Data interpolation

A simplified inverse distance weighting approach to interpolation was taken for units with missing 2011 average temperature data across H3 scale 11 and scale 7 hexagons. This simplistic approach used built-in kRings, weighting each by the distance band and giving less weight to neighbors based on how many cells away they were, to a maximum distance of 20 cells. Due to the number of empty cells it was important to rescale each value based on the number of values at all distance bands rather than the number of cells at each distance band. The equation can be expressed as:

$$\;\text{Hex}\;\text{Value}\;=\frac{\sum_{i=1}^{n=20}\left(\frac{x_{i}}{k_{i}}\right)}{\sum \frac{1}{k}}$$

where x, the value from a given cell, is divided by k, its distance band from the hexagon of interest. The result for all neighboring cells with a value within distance bands of 1 to n, 20, is summed for the numerator. The denominator is the sum of 1 divided by distance bands used. Since all points within a distance band are treated as equidistant and each band is a multiple of that distance, the actual distance is never measured directly.

## 3. Results

### 3.1. Comparison of scales

**Fig. 3.**
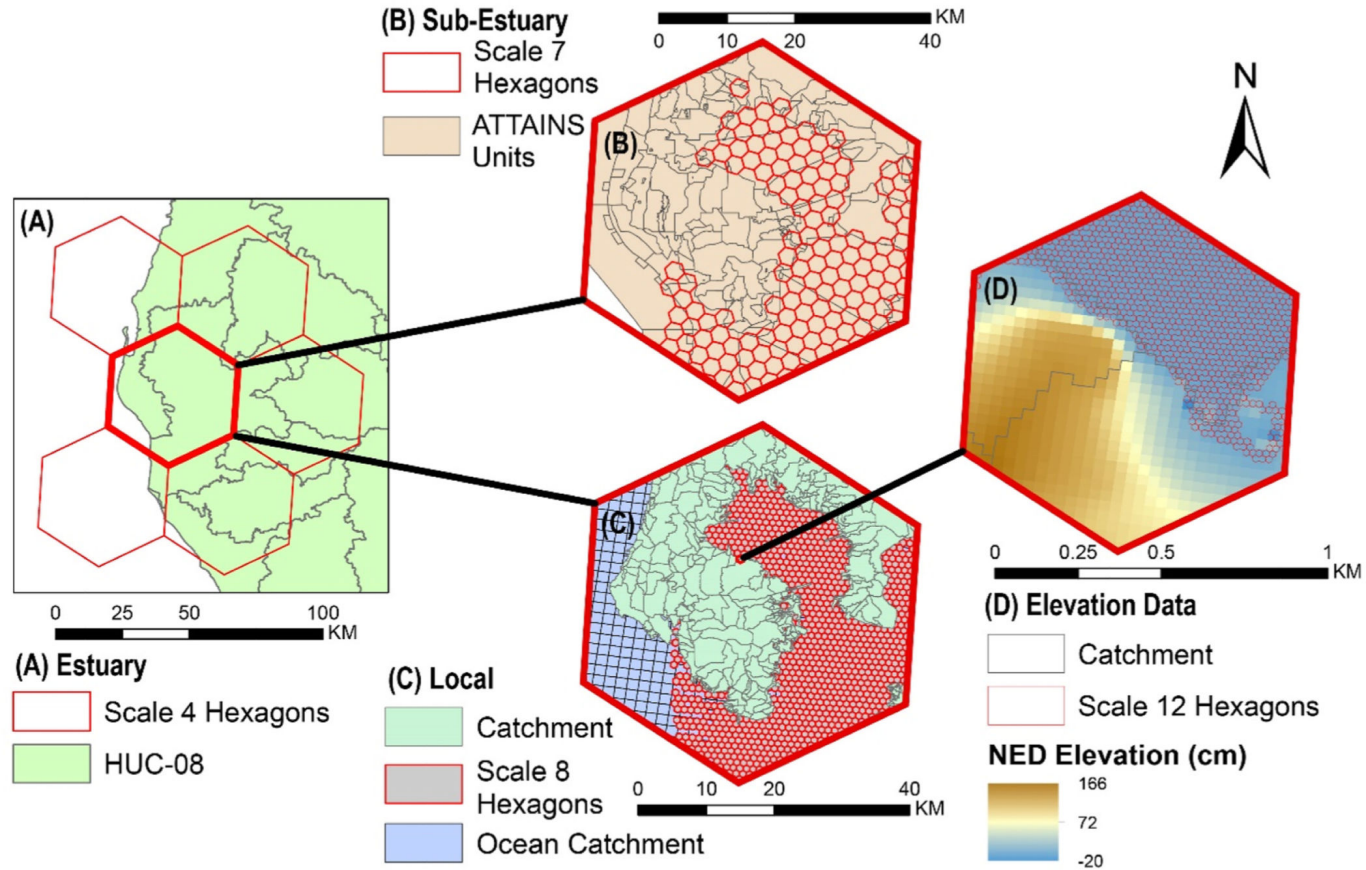
Scaled H3 hexagon unit representation against other units in Tampa Bay. (A) Scale 4 H3 hexagons compared to HUC-08, (B) Scale 7 H3 hexagons compared to ATTAINS units, (C) Scale 8 hexagons compared to NHDPlus catchments and ocean catchments, (D) Scale 12 H3 hexagons compared to 30 m NED elevation raster.

Four scales were considered from existing spatial frameworks (Table 2), NHDPlus land and ocean catchments (local), ATTAINS waterbodies (sub-estuary), WBD HUC-08 (estuary) and the elevation data resolution (Table 2; Fig. 3). The average area of H3 and dggrid hexagon units at different scales were compared to units from existing spatial frameworks at the four scales (Table 3). H3 hexagon units were mapped alongside units from the spatial frameworks at the four local scales to visualize this comparison in Tampa Bay, FL (Fig. 3).

At the local scale, comparable to 2.0/2.9 km^2^ ocean/land catchment units, H3 hexagons scale 8 to 7 have 0.7370–5.4610 km^2^ areas, respectively. dggridR hexagons scale 13 to 12 have 0.7601–3.0402 km^2^ areas, respectively. At the sub-estuary scale, comparable to 22 km^2^ ATTAINS waterbodies, H3 scale 7 to 6 hexagons have 5.1613–36.1291 km^2^ areas. dggridR scale 11 to 10 hexagons have 12.1609–48.6436 km^2^ areas. At the estuary scale, comparable to a 1800 km^2^ 8-digit HUC, H3 scale 4 to 3 hexagon areas are 1770.3240–12392.2649 km^2^ and dggridR scale 8 to 7 hexagon areas are 778.2984–3113.1935 km^2^.

Comparing the elevation data grid size to units at different hexagon scales should be based on the accuracy of those units rather than coverage area. The accuracy of a unit is based on the maximum distance between any two points in that unit. The maximum distance from any point in a hexagon to any other point in the hexagon is the width along the long diagonal, measured vertex to opposite vertex and is double edge length. For a square it is measured from vertex to opposite vertex and is √2 times the length of one side. Elevation data on a 30-m grid therefore has a 42 m accuracy and is comparable to a hexagon unit with a width of 42 m. This is within H3 hexagons scale 12 to 11, with an 18.8320–49.8220 m accuracy. For dggridR, it is within hexagons scale 18 to 17, with a 33.8047–67.6094 m accuracy. In other words, H3 scale 12 hexagons with a width of 18.832 m are comparable to a square unit 13.316 m wide, whereas H3 scale 11 hexagons are comparable to a square unit 35.235 m wide. The highest scale H3 hexagon is 15, with a 0.9 m^2^ area and an accuracy comparable to a square unit 0.7212 m wide. Due to its less restricted indexing, dggridR has a maximum scale of 30 with the projection and aperture explored here. At scale 30, hexagons have a 0.4424 cm^2^ area, comparable to a square unit 0.5835 cm wide.

The intent of these scale comparisons is to represent a similar level of aggregation. Section 3.4 explores point data converted to H3 hexagons for potential data scale distortion when moving between scales without re-aggregating.

### 3.2 Networking frameworks

**Fig. 4.**
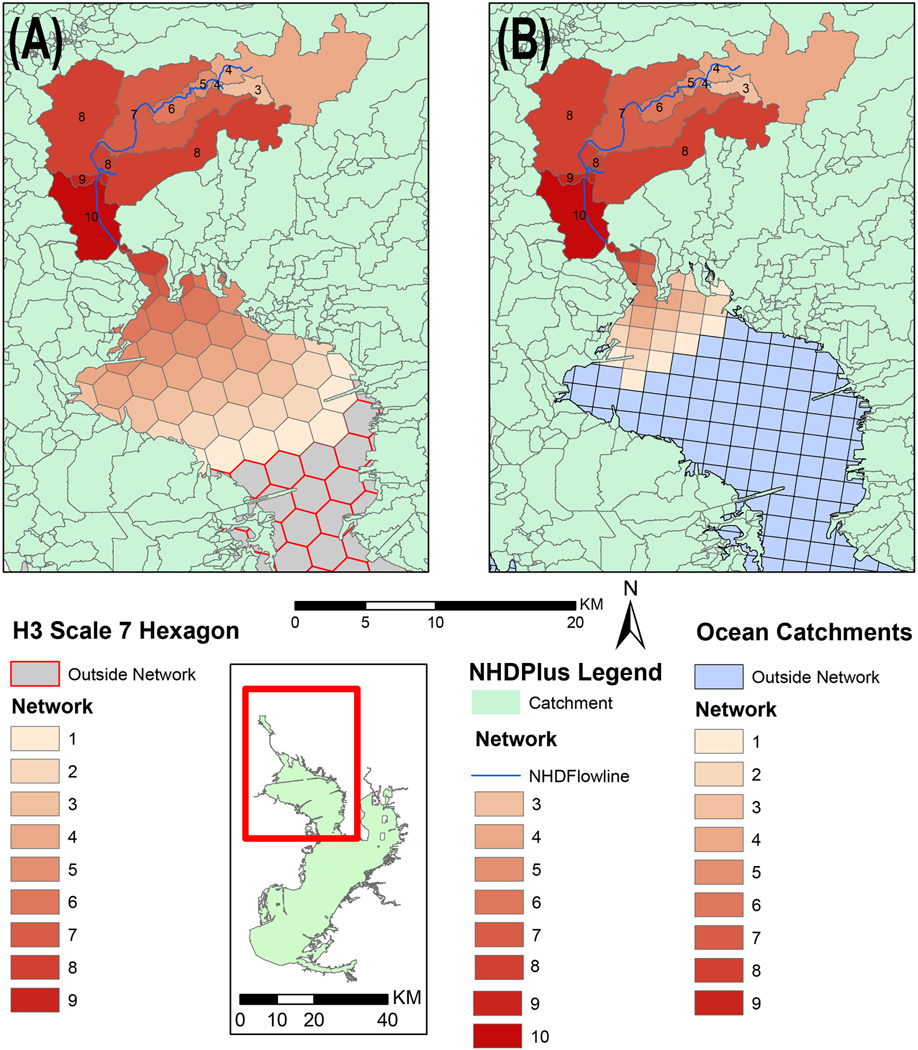
Color coding represents distance from catchment discharge point (A) networked across H3 scale 7 hexagons (B) networked across ocean catchments.

Ocean catchments and H3 hexagons were added to the flow network as downstream of land catchments. Within ocean catchments and H3 hexagons, adjacent neighbors were also networked. For ocean catchments these were non-diagonal neighbors. To visualize this network structure, a field was added to land catchments and one coastal catchment was populated with a value of 10. Using the network, upstream/ downstream spatial units were given a value of 10-n, where *n* is the number of units away from the source to the unit. This helps to quickly show how neighboring units relate (Fig. 4). The relationship is simple, but the structure allows for more sophisticated relationships between sources in upstream land catchments and downstream coastal hexagons.

### 3.3. Data aggregation

**Fig. 5.**
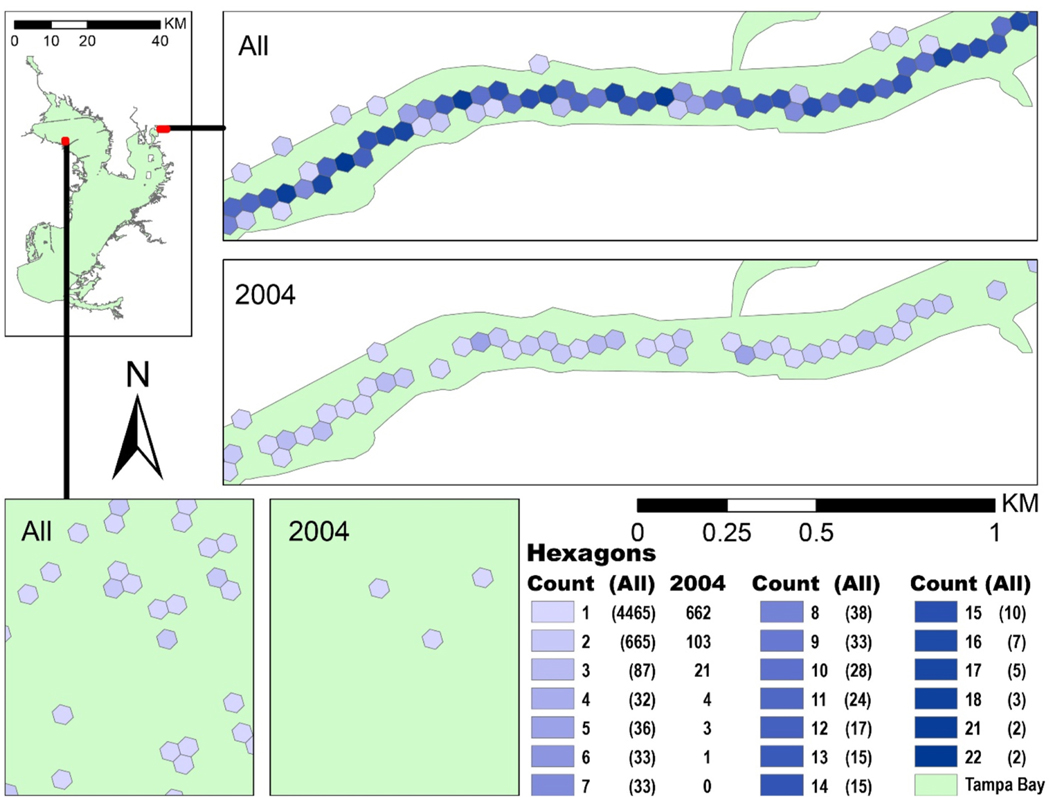
Sample point counts across scale 11 hexagons, focused in on two areas and across two time periods. ‘All’ represents hexagons for sample points (5550 total) in all 26 years. ‘2004’ represents hexagons for sample points (794 total) in 2004, the year with the most results.

Using the extent for the Tampa Bay area, WQP returned 12,356 unique sample sites. Of those sites, 9034 were within the NCCA-defined Tampa Bay area. Aggregating these site points to the H3 scale 12 hexagons resulted in 6554 hexagons. The maximum number of sites in one hexagon was 12. Aggregating to H3 scale 11 reduced this number of hexagons to 5550, with a maximum site count of 22 at two hexagons (Fig. 5).

Over the 26-year period of data retrieved (1–4-1995 to 12–21-2020), there were 182,418 water temperature results at the sample sites. This number was reduced to 170,161 water temperature results after pre- processing, with 1–5698 results at each of 5550 sites. Aggregating to daily averages reduced the total to 54,756 results, across 3987 unique days (42% of the 9480 days retrieved). Aggregating to annual averages reduced results to 14,012. Most sites (92%) only had results in one year. The year 2004 had the most sites with temperature results with 883. The 2004 average temperatures aggregated to 794 hexagons, with a maximum of 6 sites in a single scale 11 hexagon (Fig. 5).

**Fig. 6.**
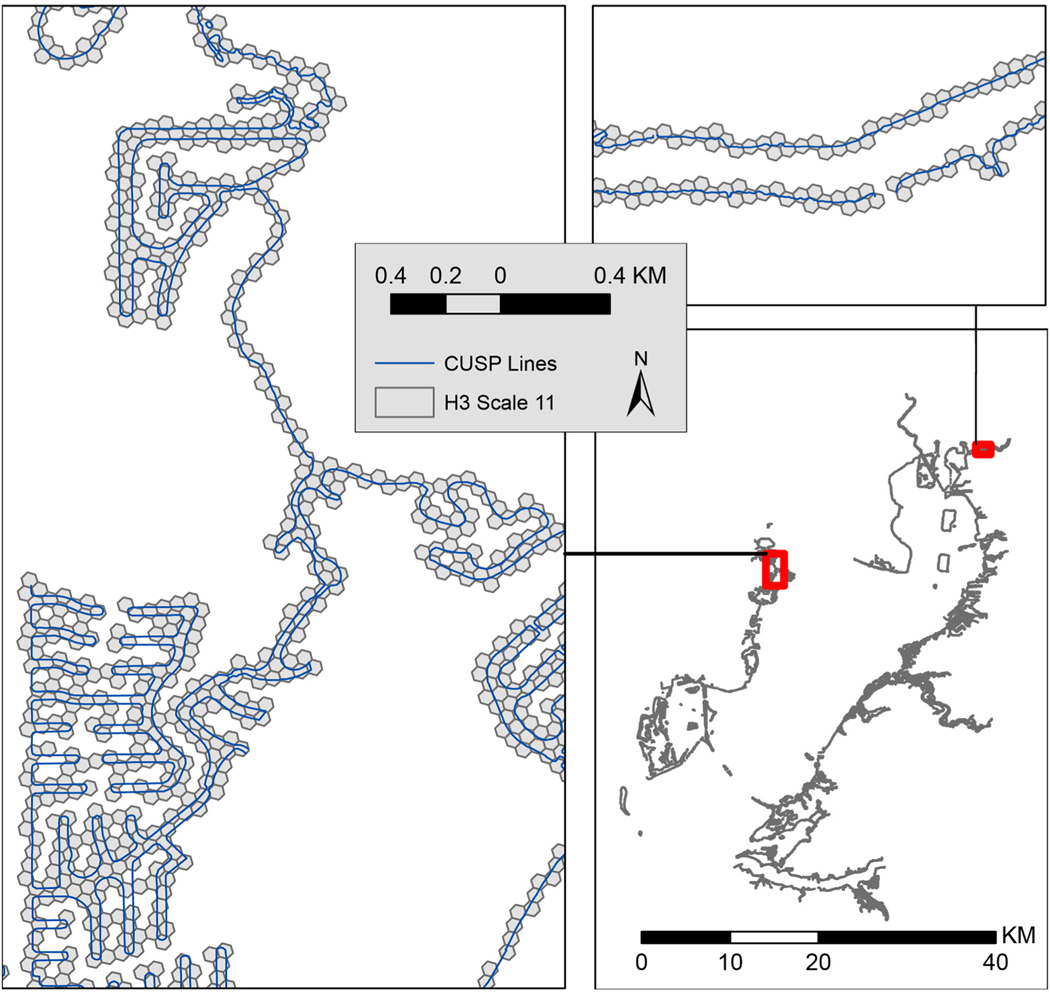
CUSP line converted to scale 11 hexagons.

The results from converting NOAA’s CUSP data to hexagons were more convoluted than anticipated. Each vertex is converted to a hexagon index, meaning sub-hexagon scale intricacies such as dips or double backs are lost at course scales. The set of hexagon indices that make up each CUSP line can be treated either individually – to allow for identification of multiple parts of the shoreline within single hexagons, or combined – to identify the unique set of hexagons intersecting CUSP lines in aggregate. The latter approach was employed as it is much easier to visualize (Fig. 6).

**Fig. 7.**
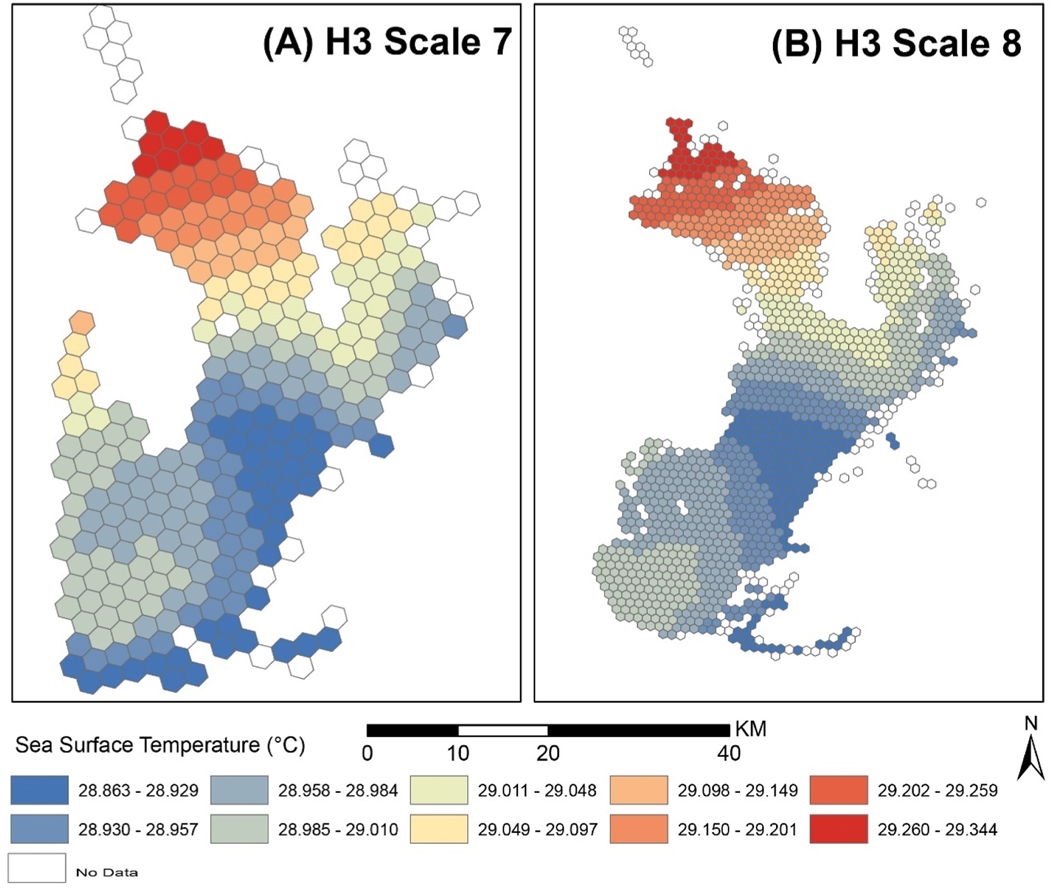
Gridded Multi-scale Ultra High Resolution Surface Temperatures (oC) for June 1, 2019 aggregated to (A) H3 scale 7 hexagons, and (B) H3 scale 8 hexagons.

The spatial resolution of the gridded data was 1-km, between H3 scales 7 and 8. The GeoTiff was converted to a comma-separated-values (csv) file with coordinates and values and the points were aggregated to scale 7 hexagons. This has the same effect as a fishnet with points at raster cell centroids. Scale 7 hexagons were larger than the gridded data resolution, and each hexagon value is the average of the point values within it. This may distort aggregate values slightly compared to an area-weighted average, but should be comparable to nearest-neighbor resampling. Scale 8 hexagons are smaller than the grid resolution and the same method would create gaps anywhere a hexagon fell between raster centroids. To avoid this the grid data was instead sampled using scale 8 hexagon centroids (Fig. 7).

### 3.4. Data scaling distortion

**Fig. 8.**
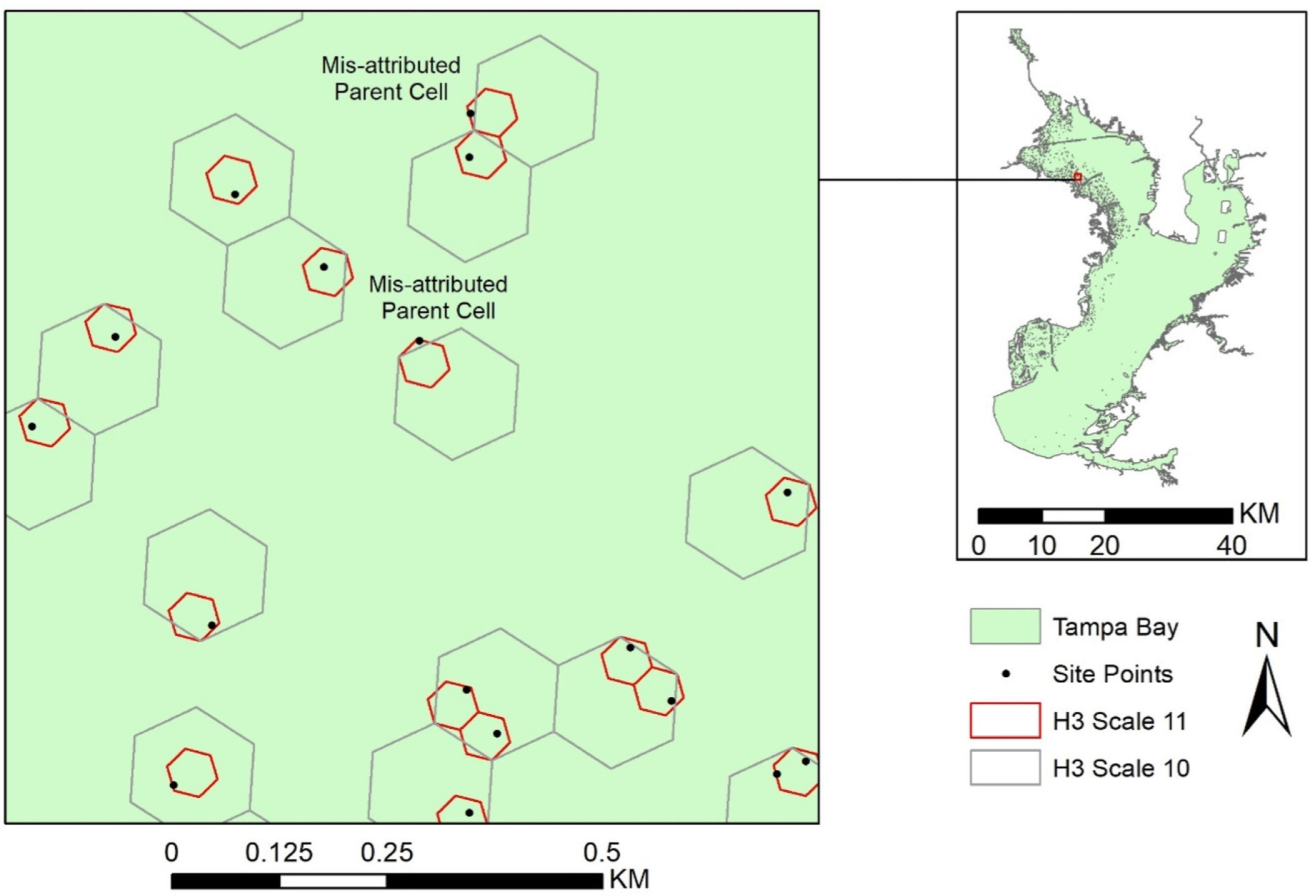
Example of data point that would cause scaling distortion.

A global spatial system faces several spatial distortion sources. One source is the non-congruence of the aperture-7 hexagon units between scales when data is scaled by truncation, as it allows data to be assigned to a parent hexagon that does not overlap the original coordinate point (Fig. 8). The Tampa Bay demonstration identified examples of this scaling distortion between scale 11, with 2293 hexagons, and scale 10, with 2904 hexagons. Only 97 hexagon assignments changed when using index-based scaling (h3_to_parent) compared to reconverting sample points to scale 10. This represents a small portion (5%) of the 1904 hexagons at scale 10. This comparison did not account for sites that may have been incorrectly assigned to a parent cell already containing a different sample site. Based on the geometry, 14.375% of the area of a parent scale unit is non-congruent with units of a child, with 7% of the child unit area outside the parent unit and 7% of parent unit area outside the child units. It is important to note assigning coordinates a new index at a new scale avoids this distortion.

Index-based scaling is not an option for the aperture-4 hexagons generated by dggridR, as scaling distortion would be greater. Their more regular geometry, with half of each exterior child unit outside of the parent unit, leads to ~43% of child cell areas being outside of the parent unit, but no points within the parent unit that are not in one of the child units. Identifying points in aperture-4 units with multiple parent units for selective re-indexing is computationally feasible. However, re-indexing is computationally efficient, making it more reasonable to just re-index all aperture-4 parent units.

### 3.5. Data interpolation

**Fig. 9.**
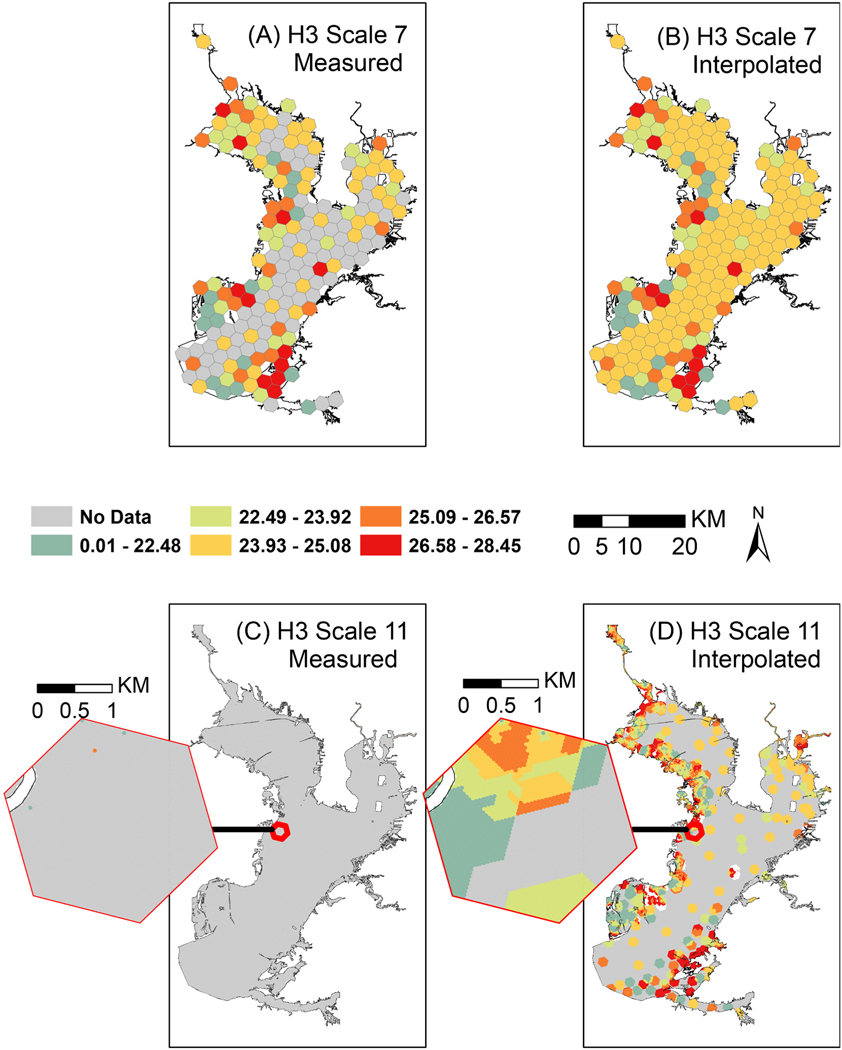
2004 measured (left) and interpolated (right) Water Temperatures across Tampa Bay Area at H3 Scale 7 (top) and Scale 11 (Bottom). Maps showing H3 Scale 11 hexagons (C & D) include an inset zoomed in on a Scale 7 hexagon to highlight the considerably smaller units and impact on both (C) aggregation of measured values and (D) interpolation.

The available water temperature point data were aggregated to hexagons but there are many hexagons within Tampa Bay that do not have temperatures sampled. The equidistant network of hexagons and built-in Hex_ring (hexRange) and k_ring (kRing) methods make simple distance weighted linear interpolation of these hexagon values efficient (Fig. 9). Though this interpolation is simple, the same built-in methods can be used with more advanced machine learning methods.

## 4. Discussion

The two hexagonal implementations, H3 and dggridR, were both able to represent data at similar scales to the existing frameworks explored here (NHDPlus land and ocean catchments, ATTAINS waterbodies and WBD HUC-08). For this application, the level of approximation achieved by both hexagonal frameworks was adequate given that it was well within the range of areas for irregular units, such as catchments. Both a larger and smaller hexagon resolution were identified for each decision scale, leaving options for end users to tailor to their specific context. If matching unit area more closely is important, dggridR hexagons of mixed 3 and 4 aperture can be used. Though not available in dggridR, DDGRID does this with preset grids (e.g. approximates a 500 m grid with the “superfund” preset; Sahr, 2019a).

Both implementations have scales able to represent the original data used to define the other frameworks, i.e. the NHDPlus raw data resolution. These scales were compared based on the accuracy of raw data rather than the unit area, i.e. 42 m for the 30 m grid. The same scales also exceed the sample point accuracy, +- 0.2 Nautical Miles (+- 37 m), required of NCCA methods (USEPA, 2010). Although hexagon scales compared favorably to existing framework units, hexagons at these scales would not represent the same spatial relationships between units. To use hexagons this way, the raw elevation data would need to be aggregated to hexagons and then hexagon-based catchments could be delineated. Liao et al. (2020) used a hexagon grid to delineate watersheds and found improvements over conventional square grids, suggesting the same could be done using H3 or dggridR.

The most resolute scale used in H3 (scale-15, 0.9 m^2^ unit area) is more resolute than similar frameworks in development, such as NHDPlus HR (Buto and Anderson 2020), and even higher resolution land use land classifications such as NOAA’s 1 m^2^ Coastal Change Analysis Program (McCombs et al., 2016). However, this is not enough to compare to the accuracy of some data collection efforts, such as individual lidar point clouds, and with advances in data resolution over the past decade it is conceivable that H3 scale-15 may become inadequate for some applications in the future. dggridR is less limited by the index system used and therefore can achieve smaller scales than H3, down to a scale-30 with 0.8253 cm unit area. By tweaking the aperture and projection, even smaller units could likely be achieved.

This application benefitted from H3 index methods, but there may be other use-cases where dggridR is superior. H3 Aperture-7 indexing was more computationally efficient compared to dggridR SEQNUM assignment. H3 built-in index-based neighbor functions were used for networking flows between H3 units, whereas networking dggridR units required storing neighbors in a PlusFlow like table. Since coastal flows were assumed to be bi-directional instead of one-way like upstream to downstream, this flow table gets larger faster and could be prohibitive at large scales. Some of the built-in index-based functionality in H3 could be added to dggridR, but would require customization for each aperture, projection, and typology. Some of this functionality may be available in newer versions of DGGRID not yet used by dggridR. Without the full suite of DGGRID options, such as the superfund preset grid, dggridR lacks H3’s simplicity of navigation between grid resolutions and neighbors. Compared to H3 dggridR has more options for how hexagon units are defined (i.e. aperture, projection, typology), can achieve smaller scales (30 vs. H3’s 15), and can use mixed 3 and 4 apertures to better approximate unit areas. This makes dggridR a better implementation for applications requiring hexagon units that are very small, in a specific area, or otherwise need to align in a specific way.

H3 had a clear advantage over dggridR or ocean catchments when networking neighboring units, determining neighbors from the unit index directly rather than needing to store it in a separate table. This allows omnidirectional networking, but more sophisticated connectivity would have to be stored in a different way. One option not explored here is to use alternative H3 index modes. Modes are 4 bits of the 64-bit integer, where mode 1 is the cell index, mode 2 is a unidirectional edge index (e.g. unit 1 → unit 2), and mode 3 will be a bi-directional edge index (e.g. unit 1 ←→ unit 2; Uber 2020). Although mode 3 is still in development, this could be an area for future exploration.

In addition to networking hexagonal units together, another objective was to network the units to other frameworks. Networking to NHDPlus catchments was of particular interest given these could act as upstream sources of downstream water quality impairments. To connect any of the units to land catchments the unique ID was added as downstream to neighboring land catchments in the PlusFlow table. This worked well for both ocean catchments, since they already have a 9-digit index unique from any land catchments, and for H3 indexes, since they all have more than 9 digits. Given the sequential dggridR SEQNUM assignment, the risk of duplicating an NHDPlus index is much higher depending on the region being considered. In Tampa Bay, the number of dggridR units would have to be considerable (25,000+) for this to be an issue. One way to avoid the issue would be to alter the stored index, for example making it a 9-digit index not already occupied by land catchments (e.g. add 500000000, making index 1 into 500000001).

Once hexagons or ocean catchments were networked to land catchments, the functionality of the network changed slightly. Land catchments are upstream/downstream of one another and one catchment may have multiple catchments upstream or downstream. Stepwise navigation – incrementally querying the next upstream or downstream catchment(s) – can be chained, for example to exhaustively identify all catchments upstream or downstream in that watershed. Stepwise navigation still works with ocean catchments or hexagon units, but these units are interconnected with one another and all watersheds causing an exhaustive search to yield all catchments instead of one watershed. Land catchments are also associated with stream segments that have a distance, allowing the network to perform distance-based navigation. For example, the network could determine what catchments are within 5 km upstream. Although distance-based navigation using the expanded network still works for land catchments, ocean catchments and hexagon units do not have a similar flow path to use this functionality. The distance from one unit to the next could instead be measured as the distance from centroid to centroid, although the result is zig-zag routing from unit to unit that measures Manhattan distance rather than the shortest navigable path.

This exploratory work did not investigate spatial overlap issues with integration. Coastal hexagons were defined by NCCA estuary boundaries rather than areas missing from land catchments, and they were left overlapping with land catchments rather than clipping them to be seamlessly adjacent as ocean catchments are. Changing this would not alter how H3 hexagon indexes are assigned when filling the area of interest. When assigning raw point data to hexagons, that data could be reduced to the area not covered by land catchments to avoid double assignment. The geometry of the hexagons could be clipped to the outline of land catchments, like partial ocean catchments, when making area-based calculations and for visualizations. A similar clipping approach could be taken to identifying hexagons that are split into sub-units by land catchments. Although these hexagon sub-units share an index, flow between them may be inhibited by land. This is not always true since land catchments often extend into submerged areas. Ideally hexagon-based elevation models could be used to develop hexagon- based catchments and then those would be seamlessly adjacent. In such a framework, units at the land-sea interface could be identified as such using CUSP, as was done here, or polygon-based shoreline definitions, such as Open Street Map’s (OSMCoastline, 2021). The main advantage of polygon-based definitions is that aquatic and terrestrial areas can be discerned.

Explored scales were adequate for aggregation of point, line and grid data. The number of sample points in overlapping H3 scale 11 hexagons suggests this is an adequate scale for differentiating most sites in estuaries. When multiple sites fell within the same hexagon, the sites tended to have different sampling times, which suggests that the sampling sites were either moving over time or are intensive limited duration studies. Temperature data in Tampa Bay was investigated because it is a commonly measured characteristic and Tampa Bay is data rich, suggesting hexagon scale 11 should be adequate in other estuaries with less data as well. As coastal water quality monitoring devices become more affordable and report readings with greater frequency H3 scale 11 may become too large, but smaller alternatives are readily available down to scale 15.

Although scale 11 was adequate for most CUSP lines, these lines presented some interesting issues. CUSP lines are discontinuous, meaning they are not suited for determining what percent of the hexagon area was land or water based. Polygons, such as those available from OpenStreetMap coastline database, are better suited for that and merit future investigation. When CUSP lines were compared to land catchments, it showed these catchments do typically extend into the water, but it was difficult to consistently measure how far because CUSP lines are often close to each other to represent small channels.

Although similar raster data equivalents now exist for hexagons (de Sousa and Leitao, 2018), national datasets in the gridded raster format are far more prevalent. Raster images representing remote sensed temperatures were aggregated to H3 hexagons scale 7 and 8. Following the existing best practices and workflows (Ion et al., 2014; Robertson et al., 2020) reinforced that these scales were most appropriate. At scale 8, some hexagons covered multiple grid cells and aggregating by hexagon centroid would cause under-sampling (grid cells not being included), so they were sampled by grid cell centroid and averaged per hexagon. At scale 7, the space between some grid cell centroids exceeded the width of hexagon units. To deal with this, the grids were aggregated by hexagon centroid. Temperatures over land were excluded from analysis, but land also interferes with some of the remote sensing methods included in the dataset which may impact near coastal data accuracy (Chin et al., 2017). Future efforts to fill gaps at unsampled sites will benefit from considering remotely sensed data but should consider alignment of resolutions and the level of interpolation already present.

Scale distortion was present in Tampa Bay when using built-in index- based methods to go from scale 11 to scale 10 hexagons. This distortion occurred in a limited number of situations and, at least at scale 11, reconverting sample points to hexagon indices at the new scale was not time or processing intensive. Future work with a larger scope, e.g. across the Gulf of Mexico or all U.S. coasts, could benefit from using the built-in index-based scaling, especially for visualizations or other use cases where the decreased accuracy is a worthwhile tradeoff for reduced processing or storage size.

Even as water quality data increases, it will remain difficult to completely sample an estuary, making it important to be able to estimate water characteristics between sites. Here, the intention was to demonstrate how to interpolate using the H3 framework. H3 built-in methods for equal distance bands were helpful when conducting a simple Inverse Distance Weighted interpolation. At the smaller H3 scale 11 (Fig. 9D) the interpolated surface is not very smooth. This is likely due to a combination of data spatial and temporal sparsity, visible in Fig. 9C where most units had ‘No Data’, and the interpolation method used. The same built-in methods could also be used for more complex interpolation and more advanced machine learning methods (e.g. pyTorch; Paszke et al., 2019). More sophisticated hydrodynamic models could make use of H3 mode 2 and mode 3 to store directionality of flows across time periods to further calibrate such models to more realistic mixing. A 20-neighbor limit was placed on how far away to interpolate, but for actual use cases there are additional considerations. For example, spatial autocorrelation places limits on how fine a scale data should be discretized and/or interpolated at. From an efficiency perspective, smaller grid units allow for greater granularity and precision but tradeoff against the storage and computational cost. In large areas with no data, one alternative to interpolation may also be hexagon compaction, where hexagons of coarser scales are intermixed between smaller hexagons to reduce the storage and computation of that data. Compaction relies on truncation to move between scales, so it introduces shape distortion, but the reverse, un-compaction is exact. This means data could be interpolated at higher scales, then compacted for most use cases.

## 5. Conclusion

Discrete Global Grid System implementations will likely continue to evolve to meet new OGC standards. Yet, this work showed that even existing hexagon implementations are already an effective alternative to existing spatial frameworks. Two DGGS implementations were tested in a coastal area for their ability to match existing decision scales, integrate with existing spatial frameworks, and represent existing coastal water quality data. dggridR was at least as effective as existing ocean catchments and H3 had additional index structuring that facilitates data- scaling and interpolation. As coastal data increases in velocity and volume a DGGS is an appropriate way to continue to process this data while also still being able to integrate with other spatial frameworks.

## Supplementary Material

Supplement1

## Figures and Tables

**Table 1:** Aperture and area ratios of different grid systems.

System	Area Ratio	Orientation
Hexagon Aperture-3	1:3	Alternates (90°)
Hexagon Aperture-4	1:4	Constant
Hexagon Aperture-7	1:7	Rotates (19.1°)
Square	1:4	NA

**Table 2:** Scales and corresponding framework units average area.

Scale	Framework Unit	Area
Local	NHDPlus land catchment	2.9 km^2^
Local	NHDPlus ocean catchment	2.0 km^2^
Sub-estuary	ATTAINS waterbody	22 km^2^
Estuary	WBD HUC-08	1800 km^2^
Elevation Data	30 × 30-m grid cell	900 m^2^

**Table 3:** H3 and dggrdR Cell average attributes at different scales.

	H3 Hexagon Unit				dggridR Hexagon Unit			
		
	Scale	Area	Width (m)	Edge (m)	Scale	Area	Width (m)	Edge (m)
**Estuary**	**3**	12392.2649 km^2^	119621.716	59810.8579	**7**	3113.1935 km^2^	69232.0369	34616.0185
	**4**	1770.3236 km^2^	45212.758	22606.3794	**8**	778.2984 km^2^	34616.0185	17308.0092
**Sub-Estuary**	**6**	36.1291 km^2^	6458.966	3229.4827	**10**	48.6436 km^2^	8654.0046	4327.0023
	**7**	5.1613 km^2^	2441.26	1220.6298	**11**	12.1609 km^2^	4327.0023	2163.5010
**Local**	**7**	5.1613 km^2^	2441.26	1220.6298	**12**	3.0402 km^2^	2163.5012	1081.7506
	**8**	0.7373 km^2^	922.710	461.3547	**13**	0.7601 km^2^	1081.7506	540.8753
**Elevation**	**11**	2149.6 m^2^	49.822	24.9106	**17**	2968.9727 m^2^	67.6094	33.8047
**Data**	**12**	307.1 m^2^	18.832	9.4155	**18**	742.2432 m^2^	33.8047	16.9024
**Max**	**15**	0.9 m^2^	1.020	0.5097	**30**	0.4424 cm^2^	0.8253 cm	0.4127 cm
