# Supplementary material for: Discrete Global Grid Systems as scalable geospatial frameworks for characterizing coastal environments
Source: Environ Model Softw. Author manuscript; Available in PMC 2022 Dec 1. (PMC8958999; doi:10.1016/j.envsoft.2021.105210)
Supplement: Supplement1 [file NIHMS1753696-supplement-Supplement1.zip › 1-s2.0-S1364815221002528-mmc2.docx]

# Supplemental Materials

All test data and code are publicly available at:

<https://github.com/USEPA/Coastal_Ecological_Indicators>

The zip file included as supplemental materials are a copy of that repo at the time of publication. It includes 4 main jupyter notebooks:

**Tampa_getWQP.ipynb**

This notebook explores retrieval and processing of temperature data from the Water Quality Portal (WQP) for the area of interest (Tampa Bay, FL). The notebook includes code to save results either as a geoJSON or shapefile with point geometries for each temperature result. The data in the Water Quality Portal is constantly updated, meaning current query results will differ from the results at the time of this study. The data retrieved at the time of this study is included in the temperature_data folder for use with H3/dggridR in subsequent notebooks. To limit the file size this data was split into two separate files, Temperature_Stations.geojson with the sample points and Temperature_Results.csv with the temperature results. This notebook was written to limit dependencies, we highly recommend using the python dataretrieval or R dataRetrieval packages to query WQP data.

**Tampa_H3_Temperatures.ipynb**

This notebook uses python to explore aggregation of temperature data from WQP to H3 hexagons of different scales in the area of interest (Tampa Bay, FL). The notebook starts by walking through how to fill that area of interest polygon with H3 hexagons of different sizes. It then walks through aggregating water quality data to a given scale of hexagons, and how different time series aggregations result in different data density. Last, it demonstrates the use of k ring functionality to interpolate data across hexagons where sample data is lacking. This is the focal notebook and the requirements.txt file lists the dependencies required to run it.

**Tampa_dggridR_Temperatures.ipynb**

This notebook uses R to explore aggregation of temperature data from WQP to dggridR hexagons of different scales in the area of interest (Tampa Bay, FL). The notebook requires several R libraries to properly function. We recommend using anaconda to set up the R environment to run this notebook. dggridR must be installed from the tar.gz.

**Tampa_H3_Grid_SST.ipynb**

This notebook explores retrieval of MUR Sea Surface Temperature (SST) grid data for the area of interest (Tampa Bay, FL) and the two methods used to aggregate that data to either H3 scale 7 or scale 8 hexagons.
